# Biomechanics of Pediatric Manual Wheelchair Mobility

**DOI:** 10.3389/fbioe.2015.00137

**Published:** 2015-09-10

**Authors:** Brooke A. Slavens, Alyssa J. Schnorenberg, Christine M. Aurit, Sergey Tarima, Lawrence C. Vogel, Gerald F. Harris

**Affiliations:** ^1^Movement Analysis for Biomedical Innovation & Technology (Mobility) Laboratory, Department of Occupational Science and Technology, University of Wisconsin-Milwaukee, Milwaukee, WI, USA; ^2^Shriners Hospitals for Children – Chicago, Chicago, IL, USA; ^3^Orthopaedic and Rehabilitation Engineering Center (OREC), Medical College of Wisconsin and Marquette University, Milwaukee, WI, USA; ^4^Department of Biostatistics, Medical College of Wisconsin, Milwaukee, WI, USA

**Keywords:** biomechanics, manual wheelchair, pediatrics, propulsion, mobility

## Abstract

Currently, there is limited research of the biomechanics of pediatric manual wheelchair mobility. Specifically, the biomechanics of functional tasks and their relationship to joint pain and health is not well understood. To contribute to this knowledge gap, a quantitative rehabilitation approach was applied for characterizing upper extremity biomechanics of manual wheelchair mobility in children and adolescents during propulsion, starting, and stopping tasks. A Vicon motion analysis system captured movement, while a SmartWheel simultaneously collected three-dimensional forces and moments occurring at the handrim. A custom pediatric inverse dynamics model was used to evaluate three-dimensional upper extremity joint motions, forces, and moments of 14 children with spinal cord injury (SCI) during the functional tasks. Additionally, pain and health-related quality of life outcomes were assessed. This research found that joint demands are significantly different amongst functional tasks, with greatest demands placed on the shoulder during the starting task. Propulsion was significantly different from starting and stopping at all joints. We identified multiple stroke patterns used by the children, some of which are not standard in adults. One subject reported average daily pain, which was minimal. Lower than normal physical health and higher than normal mental health was found in this population. It can be concluded that functional tasks should be considered in addition to propulsion for rehabilitation and SCI treatment planning. This research provides wheelchair users and clinicians with a comprehensive, biomechanical, mobility assessment approach for wheelchair prescription, training, and long-term care of children with SCI.

## Introduction

Of the ~10,000 individuals who sustain a spinal cord injury (SCI) each year in the United States (U.S.), 3–5% occur in individuals younger than 15 years of age and ~20% occur in those younger than 20 years of age (Vogel et al., 2004). An estimated 1455 children are admitted for SCI treatment to US hospitals each year (Vitale et al., 2006; Riddick-Grisham and Deming, 2011). SCI often occurs as a result of an accidental injury or traumatic event and may result in physical limitations that can affect functional mobility. Individuals with SCI are often reliant upon wheelchairs for mobility and contribute to the 3.7 million wheelchair users in the U.S. (Brault and U.S. Census Bureau, 2012). Among children under the age of 18, the wheelchair is the most widely used assistive mobility device impacting over 88,000 children, 90% of which use manual wheelchairs (Kaye et al., 2000). In adults with SCI shoulder pain and degenerative changes, especially at the acromioclavicular joint, may develop prematurely due to overuse and altered mechanical stresses, particularly in those with high levels of manual wheelchair activity (Lal, 1998; Mercer et al., 2006). Reported upper extremity injuries associated with manual wheelchair usage in adults with SCI include destructive shoulder arthropathy, degenerative arthritis of the shoulder and elbow, rotator cuff tendonitis, coracoacromial pathology, and carpal tunnel syndrome (Pentland and Twomey, 1991; Sie et al., 1992; Lal, 1998; Ballinger et al., 2000; Boninger et al., 2001; Mercer et al., 2006; Yang et al., 2009). It has been reported that adult manual wheelchair users with SCI have a high prevalence of shoulder pain and injury (Boninger et al., 2001; Mercer et al., 2006; Schnorenberg et al., 2014) with shoulder pain occurrence in paraplegics ranging from 30 to 73% (Pentland and Twomey, 1991; Sie et al., 1992; Boninger et al., 2002, 2005; Mercer et al., 2006). Due to longer-term wheelchair use in those with pediatric-onset SCI, upper extremity pain and injuries may occur earlier in their lifespan and severely limit independence, function, and quality of life (Vogel et al., 2011). Previous work by our group has shown that the incidence of shoulder pain in adults with pediatric-onset SCI is 48–54% (Hwang et al., 2014); however, there is limited information on functional mobility and pain in those with pediatric-onset SCI.

Children who have sustained a SCI often use a manual wheelchair for functional mobility in the home, school, and community environments. Functional mobility includes propulsion, starting from a stationary position, stopping their wheelchair, and moving over various terrains (Case-Smith and O’Brien, 2013). Studies have examined adult manual wheelchair users during mobility tasks including level propulsion, ramp ascent, start and stop, and weight relief and found significantly different upper extremity joint demands across tasks (Morrow et al., 2010). However, children are not physically proportionate to adults and we cannot assume that scaling dynamics information will give an accurate representation of the true demands of wheelchair mobility. A study by Jensen confirmed changes in force and moment curves due to differences in proportionality and a redistribution of mass that occurs with age (Jensen, 1989). Although children are proportionately different than adults, with developing musculoskeletal systems, there is limited research of pediatric wheelchair mobility (Schnorenberg et al., 2014; Slavens et al., 2015). It is, therefore, vital that research address the unique biomechanics of pediatric wheelchair mobility and provide insight to the differences from adults. Despite this, current literature contains many studies that consider the biomechanics of adult manual wheelchair mobility, and few focused on the biomechanics in the pediatric population (Koontz et al., 2005; Petuskey et al., 2007; Rice et al., 2009; Schnorenberg et al., 2014). Pediatric manual wheelchair propulsion repetitively places increased load demands on the upper extremities (Schnorenberg et al., 2014), leading to a level of high concern for the development of pain and injury over the long-term duration of usage. Further insight into the biomechanics of pediatric wheelchair users is critical for ultimately preserving upper extremity function and joint integrity. More so, a deeper understanding of the relationship among upper extremity biomechanics, pain, and function is necessary. We aim to quantify upper extremity kinematics and kinetics during functional manual wheelchair mobility in children with SCI and identify their related pain and health-related quality of life.

In adults, four primary wheelchair stroke patterns, the motion the hand makes during the recovery phase of the stroke cycle, have been defined. These include (1) single looping over propulsion (when the hands rise above the handrim), (2) double looping over propulsion (when the hands rise above and then fall below the handrim), (3) semicircular (when the hands fall below the handrim), and (4) arcing (ARC) (when the hand follows the path of the pushrim) (Shimada et al., 1998; Boninger et al., 2002, 2005). Research has demonstrated that in adults, the semicircular pattern allows the user to apply force to the handrim over a greater angle and for a longer duration. These characteristics correlated to a reduction of injury risk in adults. Therefore, the semicircular pattern is the recommended technique for adult manual wheelchair propulsion (Boninger et al., 2002, 2005). However, it is important to note that there is a void of propulsion stroke pattern characterization in the pediatric population and studies supporting the recommendation of the semicircular propulsion pattern are limited to adult wheelchair users with paraplegia and were cautioned for application to other groups, such as pediatrics (Boninger et al., 2005). We will, thus, examine pediatric stoke patterns in this study.

The primary purpose of this study is to quantify upper extremity joint kinematics and kinetics of pediatric manual wheelchair users during functional manual wheelchair mobility. We will investigate three functional tasks: (1) propulsion, (2) starting from rest, and (3) stopping during propulsion. We hypothesize that three-dimensional (3-D) upper extremity joint motions, forces, and moments will be significantly different among the three tasks. We will also identify pediatric wheelchair stroke patterns during the propulsion task and evaluate pain and health-related quality of life outcomes.

## Materials and Methods

### Upper extremity biomechanical model

A custom, bilateral, pediatric, upper extremity model was utilized to determine 3-D joint angles, forces, and moments (Schnorenberg et al., 2014). This biomechanical model comprises 11 segments, including the thorax, clavicles, scapulae, humeri, forearms, and hands. The joints of interest are three degree-of-freedom thorax, acromioclavicular, glenohumeral (GH), and wrist joints; and two degree-of-freedom sternoclavicular and elbow joints. These segments are represented by strategically placing reflective markers on bony anatomical landmarks and technical locations of the subject, including the suprasternal notch, xiphoid process, spinal process C7, acromioclavicular joint, inferior angle (IA), trigonum spinae (TS), scapular spine, acromial angle, coracoid process, humerus technical marker, olecranon, radial and ulnar styloids, and the third and fifth metacarpals (Schnorenberg et al., 2014).

The upper extremity model includes novel features (Schnorenberg et al., 2014), some of which we will highlight. First, the markers defining the thorax segment are placed directly on the torso, with no indirect placement on the clavicles. This reduces the amount of error introduced when calculating the thorax joint angles due to clavicle motion relative to the thorax (Nguyen and Baker, 2004). Second, the elbow joint is modeled using a technique that does not require the use of a marker placed on the medial epicondyle, which is often obstructed and inadvertently interacts with the wheelchair. By using a single marker on the olecranon, inaccuracies and marker dropout are reduced (Hingtgen et al., 2006). Third, the model incorporates a scapula marker tracking technique developed by Senk et al. utilizing rigid body theory, which enables accurate calculation of markers placed on the TS and the IA of the scapulae despite the subcutaneous nature of scapula motion. This method captures the TS and IA scapula marker positions during a static position with precisely palpated positions. The TS and AI markers are then removed for dynamic trials and their trajectories calculated based on their position and orientation relative to the other scapula markers, which move more reliably during dynamic tasks. This was shown to be appropriate for scapular motion tracking, especially during tasks with <120–150° of arm elevation. This method has low root mean square (RMS) errors (5.4–10.3°), similar to those of the commonly used tracker (3.2–10.0°) and acromion (4.8–11.4°) methods (Senk and Cheze, 2010). Fourth, the ability to track these scapula positions allows the use of a more accurate method of GH joint center calculation. For this calculation, Meskers developed regression equations involving the positions of the scapula markers. These equations have since been updated by the International Shoulder Group (ISG) and were shown to be accurate when compared to magnetic resonance (MR) images of the actual joint center (Campbell et al., 2009). Lastly, we used pediatric appropriate body segment parameters and anthropometric measures (Jensen, 1989), specifically customizing our model to children and adolescents.

Segment coordinate systems were determined for each of the model’s 11 segments. Following ISB recommendations, the segment coordinate systems’ axes are aligned such that the *Z*-axis points laterally toward the subject’s right side, the *X*-axis points anteriorly, and the *Y*-axis points superiorly (Wu et al., 2005). The joint angles were determined by the relative motion between two adjacent segment coordinate systems, distal relative to proximal. The segment coordinate systems follow the right-hand rule with the *Z*-axis as the flexion/extension axis; the *X*-axis as the abduction/adduction axis; and the *Y*-axis as the internal/external rotation axis. A *Z*–*X*–*Y* Euler sequence is used to calculate the GH, elbow, wrist, and thorax joint angles, and a *Y*–*X*–*Z* Euler sequence is used for the acromioclavicular and sternoclavicular joint angle computation.

### Subjects

Approval from the Shriners Hospital for Children – Chicago’s Institutional Review Board (IRB) was obtained for the study. Fourteen pediatric manual wheelchair users with SCI were recruited and an assent form or informed consent form were signed by the child and/or their parent/guardian. All subjects were evaluated at Shriners Hospitals for Children – Chicago. Subjects included in this study were under 21 years of age, had a SCI diagnosis, were at least 1 year post-injury and used a manual wheelchair for their primary mode of mobility. The mean subject age was 13.7 ± 4.8 years, with an average time since injury of 5.3 ± 3.9 years. The bony level of SCI ranged from the third cervical (C3) vertebra to the tenth thoracic (T10) vertebra. Levels A, B, and C of the American Spinal Injury Association (ASIA) Classification, which grades the severity of an individual’s neurological loss, were represented. Subject characteristics are described in Table 1.

**Table 1:** **Subject characteristics for each subject and the calculated group averages and SDs**.

Subject	Age (years)	Height (cm)	Weight (kg)	Time since injury (years)	SCI (ASIA) level	SCI classification	Gender	Arm dominance
1	11.1	177.8	24.4	2.9	T9 (A)	Paraplegia	Male	Right
2	17.3	169.9	63.8	1.1	C6 (B)	Quadriplegia	Male	Right
3	16.8	183.1	63.8	1.3	C7 (B)	Paraplegia	Male	Right
4	11.8	152.4	58.5	NR	C8 (A)	Paraplegia	Male	Right
5	20.9	167.6	51.1	3.8	T10 (A)	Quadriplegia	Female	Left
6	19.5	193	93	1.5	C6 (C)	Paraplegia	Male	Left
7	7.2	121.9	26.5	5.8	C3-T1 (C)	Paraplegia	Male	Left
8	6.5	119.4	28.5	6.2	L3 (C)	Paraplegia	Male	Right
9	10.2	121.9	24.0	8.1	T4 (A)	Paraplegia	Female	Right
10	16.6	133.1	31.6	10.9	T10 (C)	Paraplegia	Male	Right
11	19.0	178.0	76.0	6.5	T9 (A)	Paraplegia	Male	Right
12	14.5	139.7	42.5	14.0	T8 (A)	Paraplegia	Female	Right
13	13.0	153.4	44.0	3.1	C8 (B)	Paraplegia	Female	Right
14	7.8	118.1	22.6	4.1	T10 (A)	Quadriplegia	Female	Right
Average	13.7	152.1	46.5	5.3				
SD	4.8	26.4	22.1	3.9				

*NR, not reported*.

### Data collection

A pain outcome, the Visual Analog Scale (VAS), and a quality of life outcome, the Short Form 12 Health Questionnaire (SF-12), were administered prior to motion analysis. The VAS was utilized since it serves as a standard outcome tool for clinical assessment at Shriners Hospital for Children – Chicago. Subjects were asked to indicate their average daily pain level by marking it on the scale with a pen, or pointing, to indicate their rating, with 0 being no pain at all and 100 being the worst pain imaginable (Wewers and Lowe, 1990). The SF-12 assessed the subjects’ health-related quality of life. Subjects were asked to respond to each of the 12 questions, which are used to calculate a physical composite score (PCS) and a mental health composite score (MCS) on a scale of 0–100, with the national norm score for healthy individuals being 50 (Office of Public Health Assessment, 2004).

**Figure 1:**
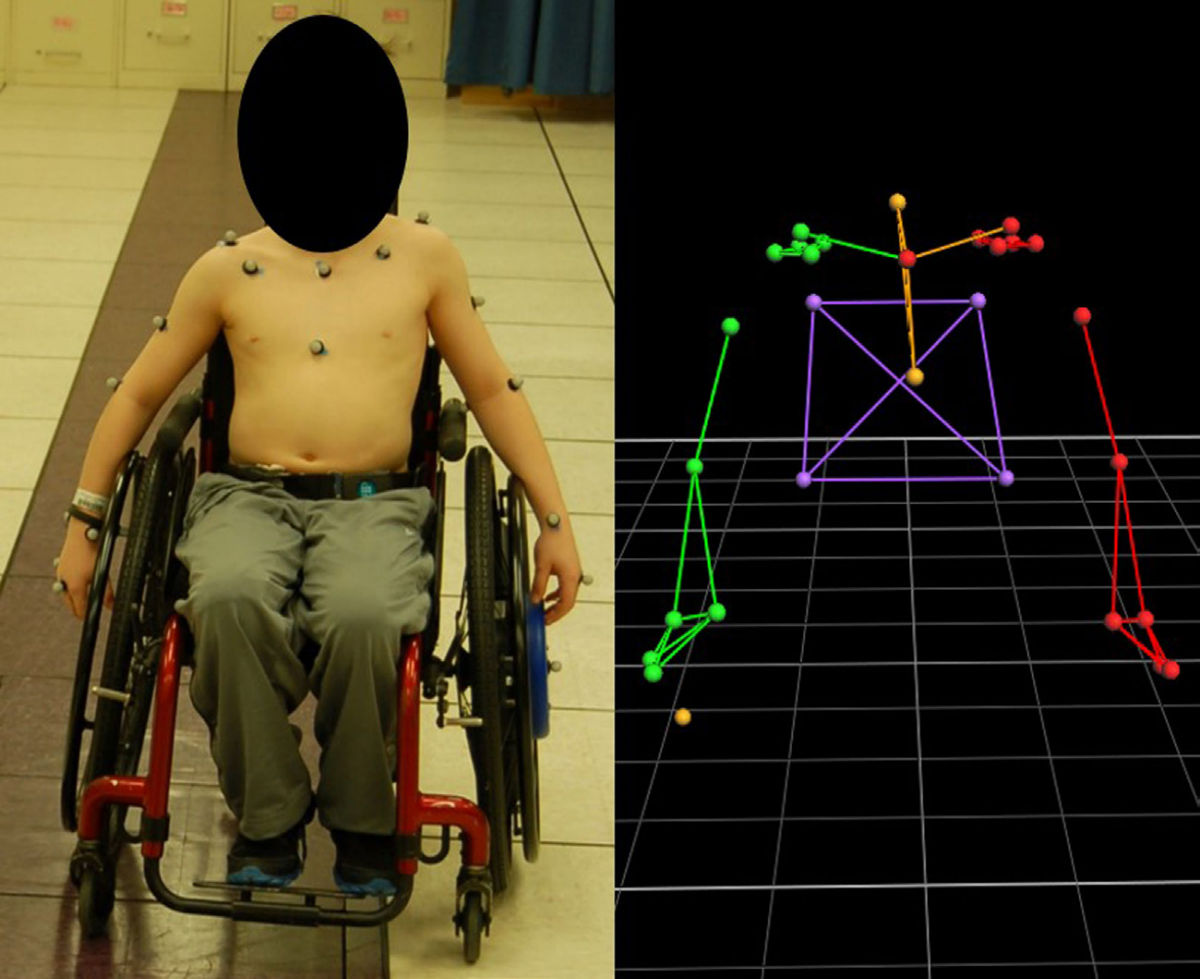
**Subject with marker set applied and SmartWheel replacing the dominant, left-hand side wheel (left) and the model rendering in Vicon Nexus software**.

Subject-specific measurements were obtained for all participants. The 27 passive reflective markers, previously described, were adhered to the subject to prepare for motion capture (Figure 1). A SmartWheel (Outfront, Mesa, AZ, USA), replaced the wheel on the dominant side of the subject’s wheelchair for kinetic data collection; the SmartWheel companion wheel replaced the subject’s wheel on the non-dominant side. Both the SmartWheel and its companion are air tires. No subject required the use of plastic-coated handrims or gloves to assist with their propulsion.

The subject propelled his or her manual wheelchair along a 15-m path at a self-selected speed and self-selected propulsion pattern to simulate community/home mobility. A 14-camera Vicon MX System captured 3-D marker trajectories at 120 Hz, while the SmartWheel simultaneously collected tri-axial forces and moments occurring at the hand–handrim interface at 240 Hz. Subsequently, the collected kinetic data was low-pass filtered using a 32-tap finite impulse response (FIR) filter. Multiple trials were collected, with adequate rest provided to the subject as needed.

**Figure 2:**
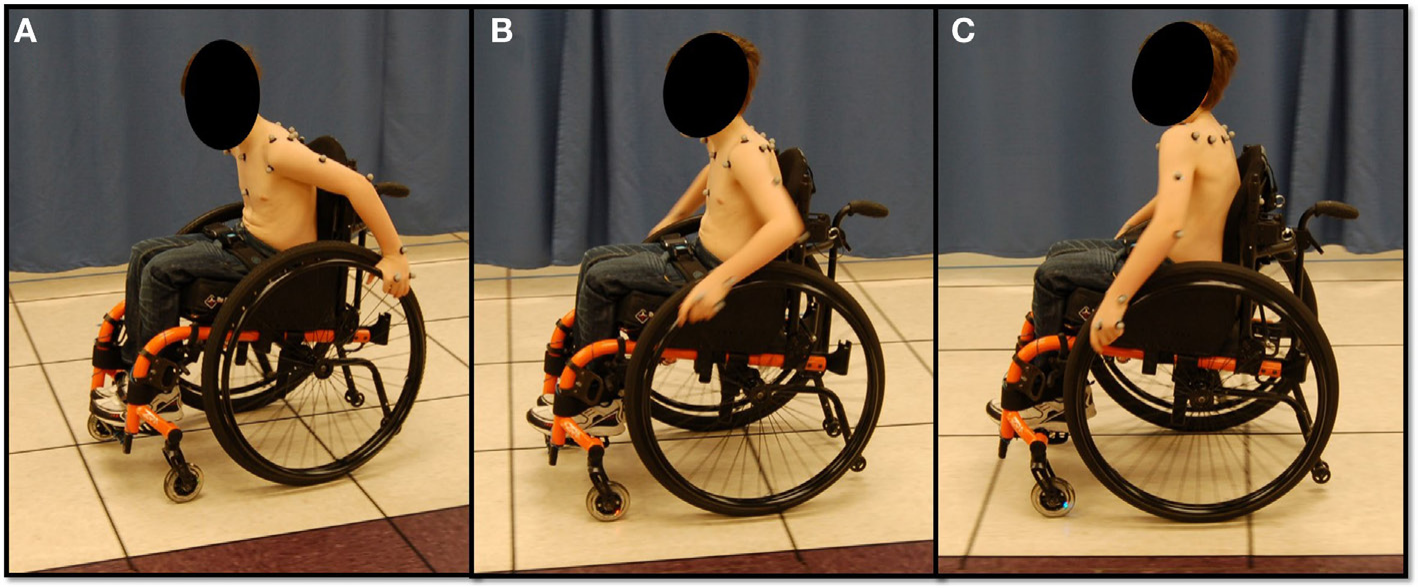
**Subject performing functional manual wheelchair mobility tasks**. **(A)** Starting, **(B)** propulsion, and **(C)** stopping.

All participants performed a series of functional mobility tasks, including propulsion, starting, and stopping (Figure 2). Propulsion involved subjects propelling their manual wheelchair across the room while staying on a colored walkway in the center of the capture volume. Ten stroke cycles, obtained from multiple trials, were analyzed. Within a trial, the start-up strokes and stopping strokes were always excluded from evaluation to eliminate effects of acceleration and deceleration. The start task began with subjects at a static position in the center of the capture volume and then propelled themselves to the far side of the room (the end of the capture volume). The first stroke was analyzed for each of the three trials. The stop task began with subjects outside of the capture volume in a static position. They then propelled themselves into the capture volume and stopped when they reached the center. The last stroke was analyzed for each of the three trials.

### Data processing

Vicon Nexus was used to process the marker trajectories. The resulting marker trajectories were filtered using a Woltring filter with a mean squared error setting of 20 (Woltring, 1986). The kinetic data was then resampled to 120 Hz in MATLAB (The Mathworks, Inc., Natick, MA, USA) to match the kinematic sampling rate.

For each subject, the wheelchair stroke cycles were analyzed to compute the mean group parameters of interest. Mean time series data of the joint angles, forces, and moments were all time normalized to the percent of the wheelchair stroke cycle. The stroke cycles were separated into two phases, contact and recovery phases, based on total force applied to the handrim, following the definitions of Kwarciak et al. (2009). The stroke pattern was determined using the sagittal plane motion of the marker on the third metacarpal, plotting the vertical position versus fore–aft position (Shimada et al., 1998; Boninger et al., 2002, 2005). Peak joint angles (maximum and minimum) were identified and used to compute angular ranges of motion (ROMs). Maximum and minimum joint forces and moments were also identified and are referred to as peak forces and moments.

### Statistical analyses

Linear mixed models (LMM) were used for statistical comparisons amongst group joint ROMs and peak dynamics separately for each task. Random subject effect was used to control for possible within subject correlation. LMM were also used to investigate statistical significance of differences in biomechanical outcomes of the joints among the tasks.

## Results

### Joint kinematics

**Figure 3:**
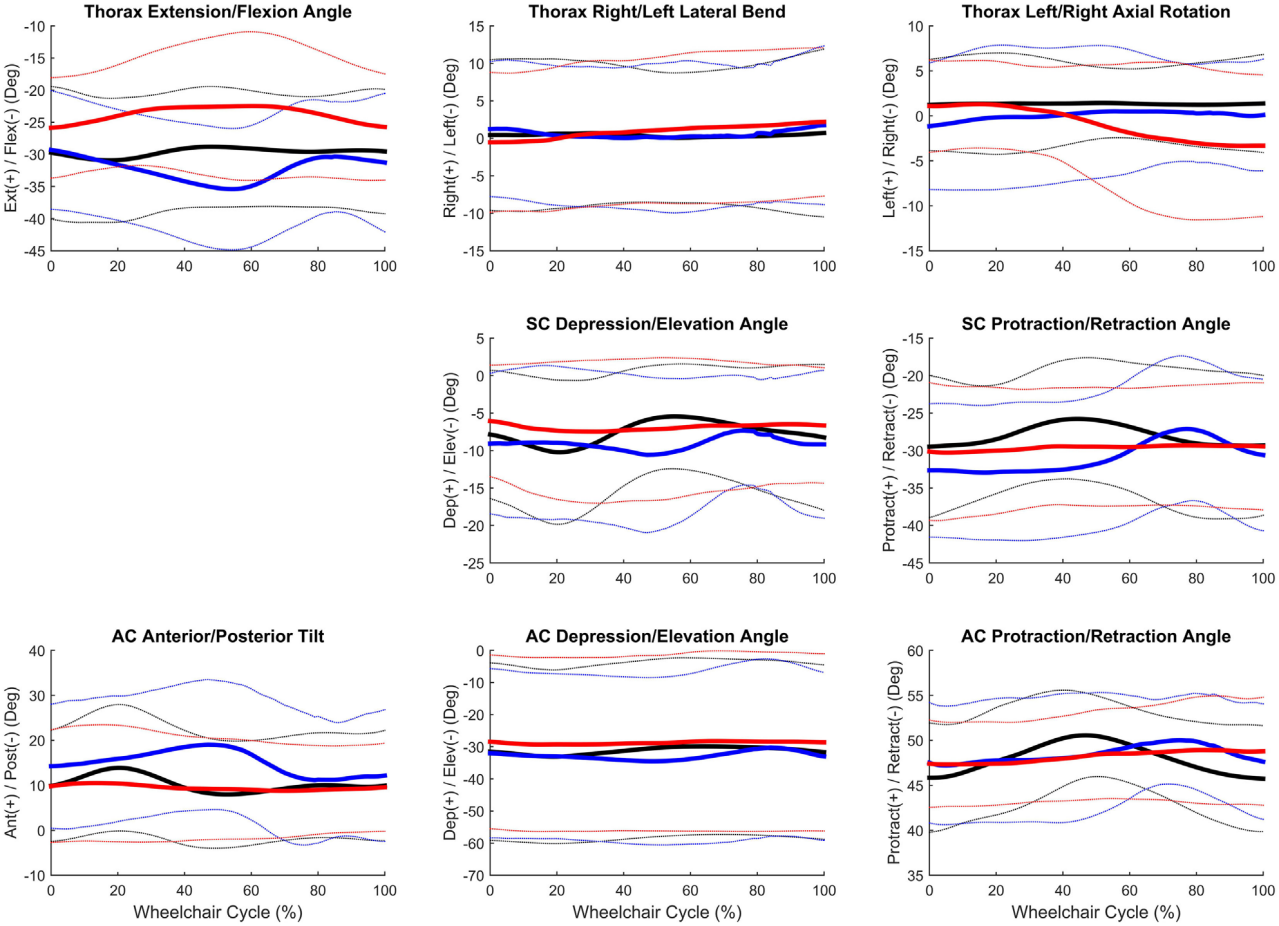
**Group mean (bold) and ±1 SD for the thorax joint angles (top row), sternoclavicular joint angles (middle row), and sternoclavicular joint angles (bottom row) during the steady-state propulsion (black), start stroke (blue), and stopping stroke (red)**.

**Figure 4:**
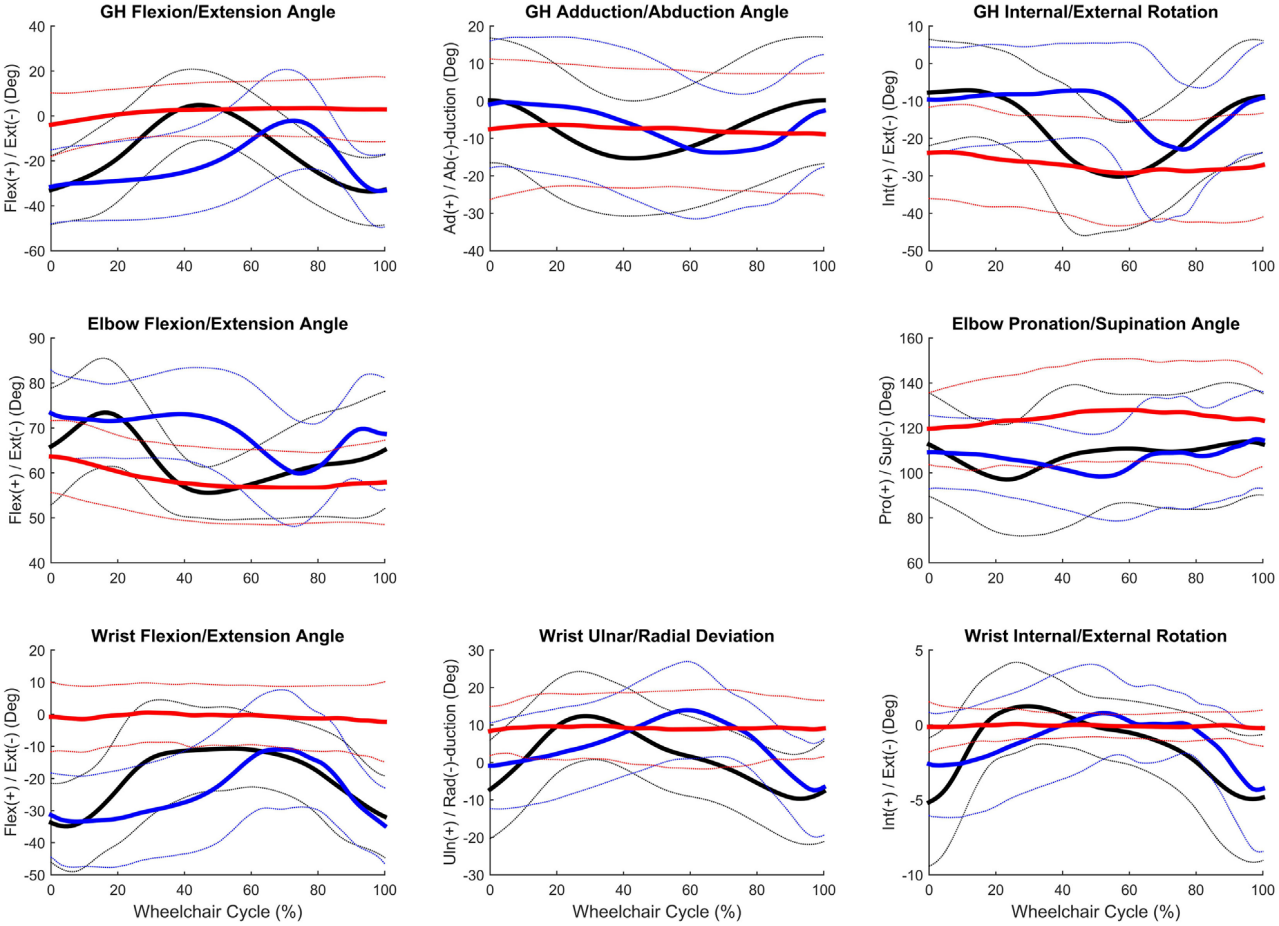
**Group mean (bold) and ±1 SD for the glenohumeral joint angles (top row), elbow joint angles (middle row), and wrist joint angles (bottom row) during the steady-state propulsion (black), start stroke (blue), and stopping stroke (red)**.

**Figure 5:**
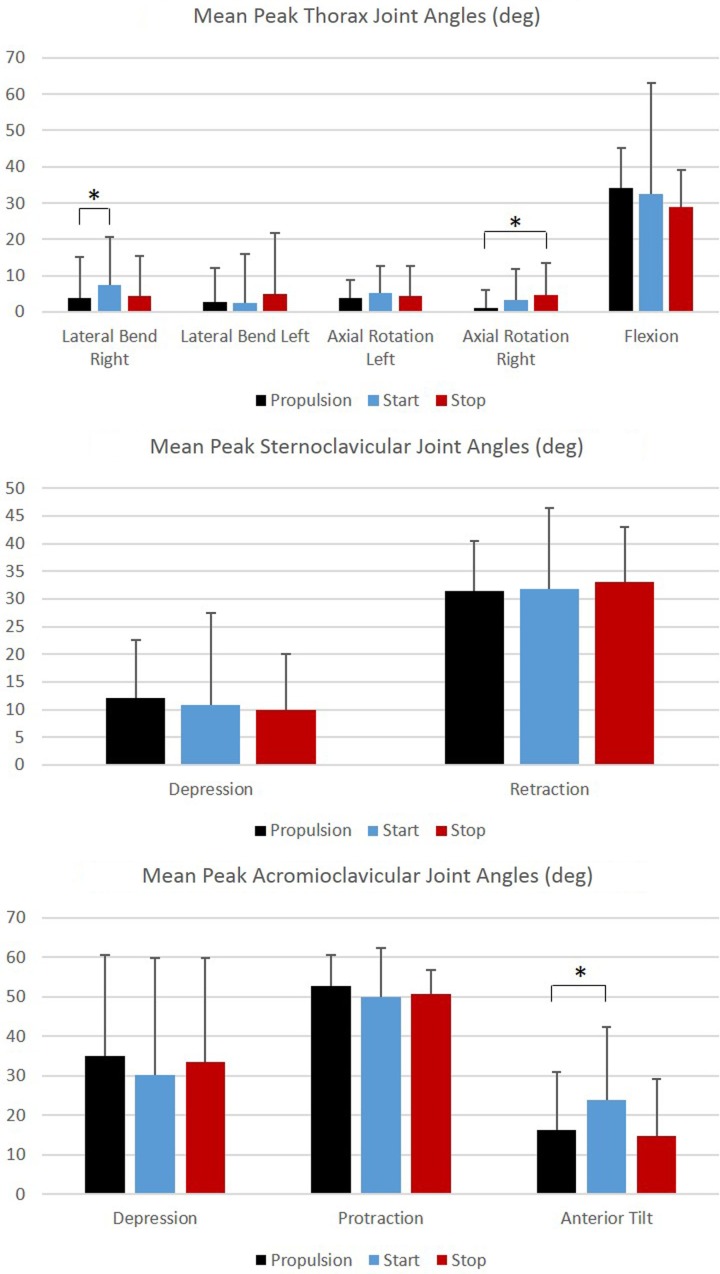
**Group mean peak joint angles (degrees) for the thorax, sternoclavicular, and acromioclavicular joints during each functional mobility task, propulsion (black), start (blue), and stop (red)**. One SD is represented by the thin vertical bar. Tasks connected by an asterisk are statistically significantly different (*p* < 0.01).

**Figure 6:**
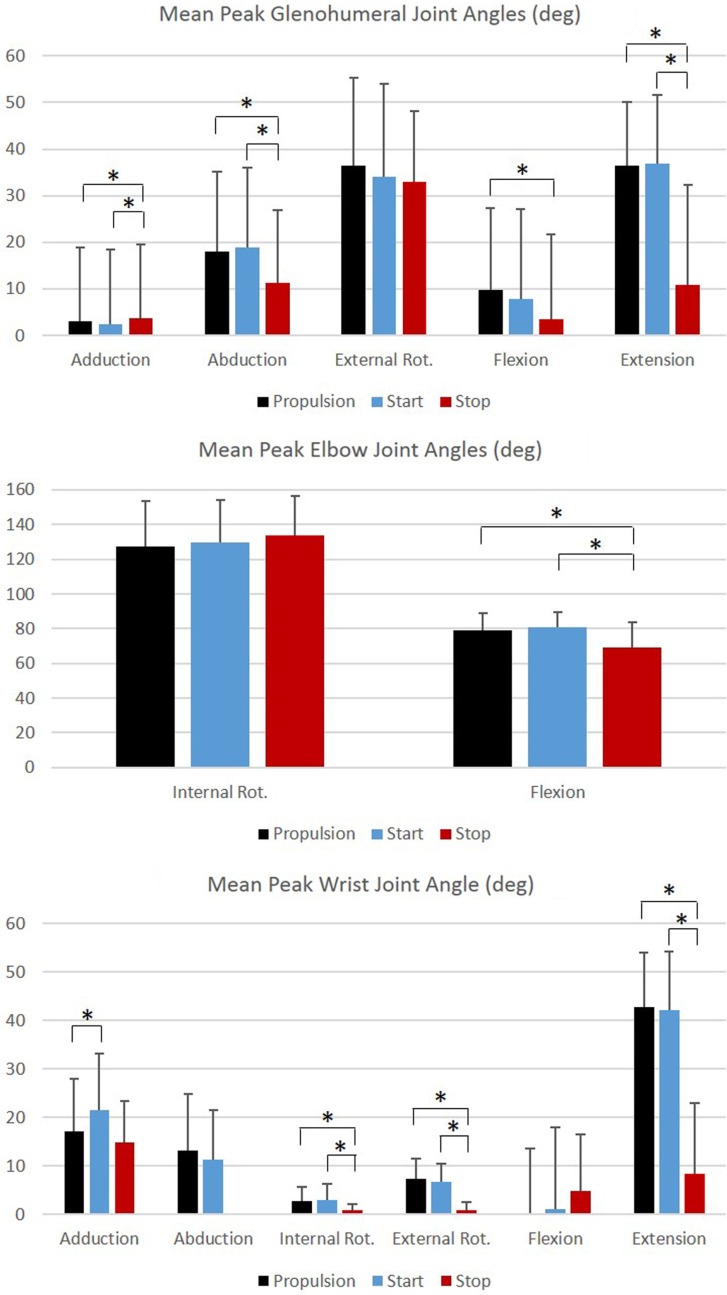
**Group mean peak joint angles (degrees) for the glenohumeral, elbow, and wrist joints during each functional mobility task, propulsion (black), start (blue), and stop (red)**. One SD is represented by the thin vertical bar. Tasks connected by an asterisk are statistically significantly different (*p* < 0.01).

**Figure 7:**
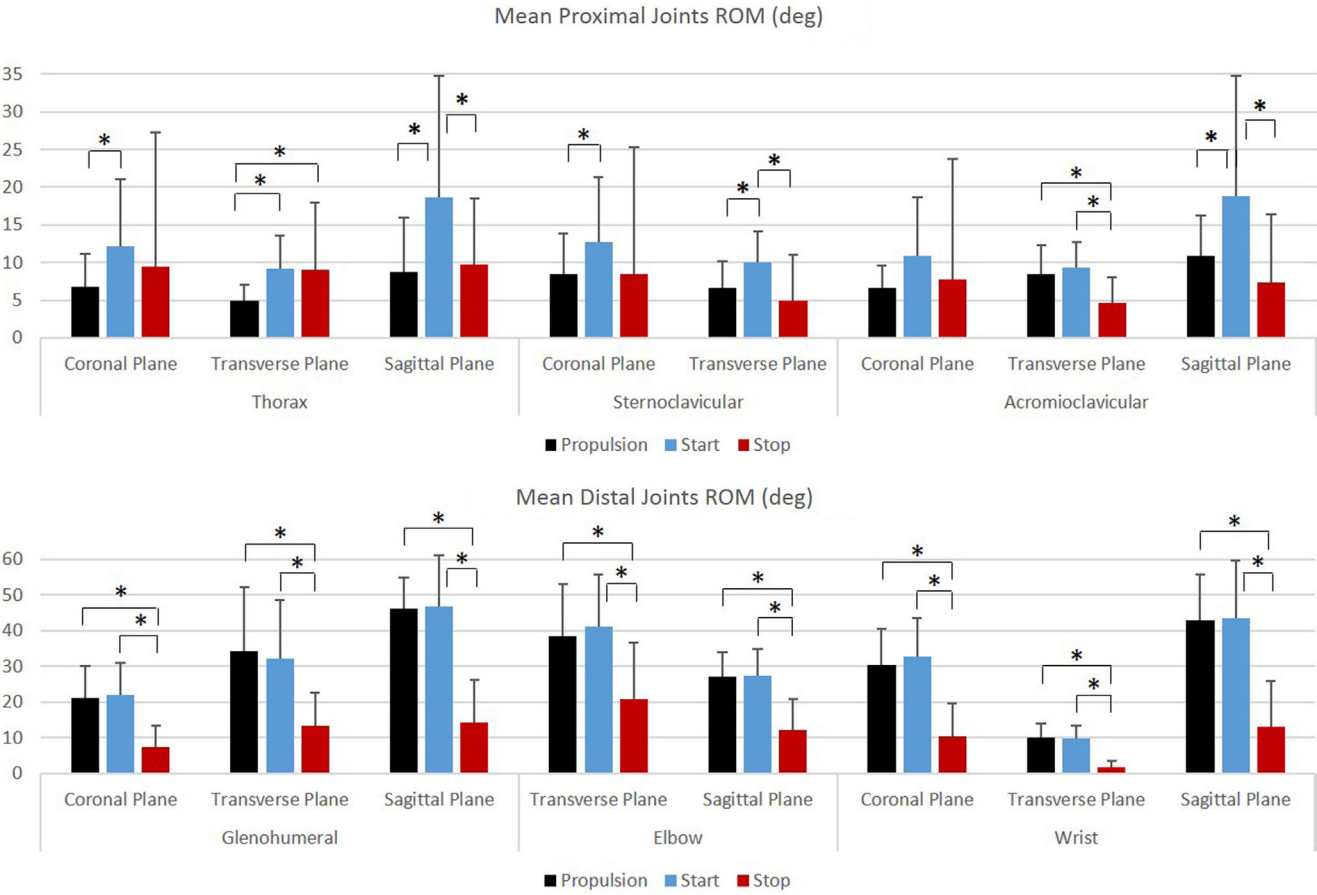
**Group mean joint ranges of motion (degrees) for the proximal joints (top row: thorax, sternoclavicular, and acromioclavicular) and the distal joints (bottom row: glenohumeral, elbow, and wrist) during each functional mobility task, propulsion (black), start (blue), and stop (red)**. One SD is represented by the thin vertical bar. Tasks connected by an asterisk are statistically significantly different (*p* < 0.01).

Group mean joint angles were characterized in all three planes of motion over the wheelchair stroke cycle for the propulsion, start, and stop tasks. The mean and ±1 SD for the thorax, sternoclavicular, and acromioclavicular joints are shown in Figure 3 and for the GH, elbow, and wrist joints in Figure 4. The mean peak joint angles (Figures 5 and 6) and mean joint ROMs (Figure 7) over the wheelchair stroke cycle were also calculated. Statistically significant differences (*p* < 0.01) in mean peak joint angles and mean ROMs among tasks were identified and are depicted in Figures 5–7.

### Joint kinetics

**Figure 8:**
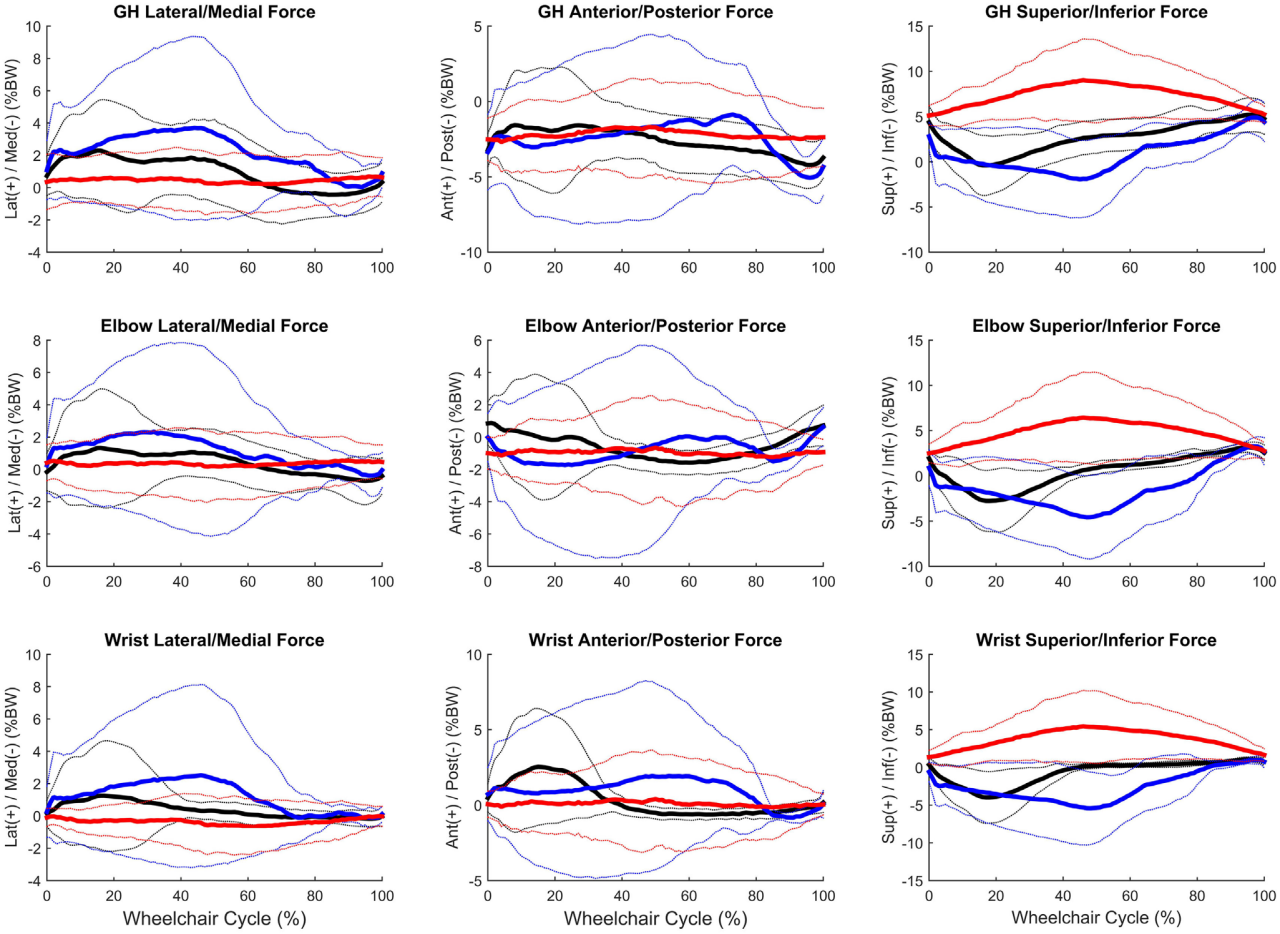
**Group mean (bold) and ±1 SD for the glenohumeral joint forces (top row), elbow joint forces (middle row), and wrist joint forces (bottom row) during the steady-state propulsion (black), start stroke (blue), and stopping stroke (red)**. All forces are normalized to percentage of body weight (% BW).

**Figure 9:**
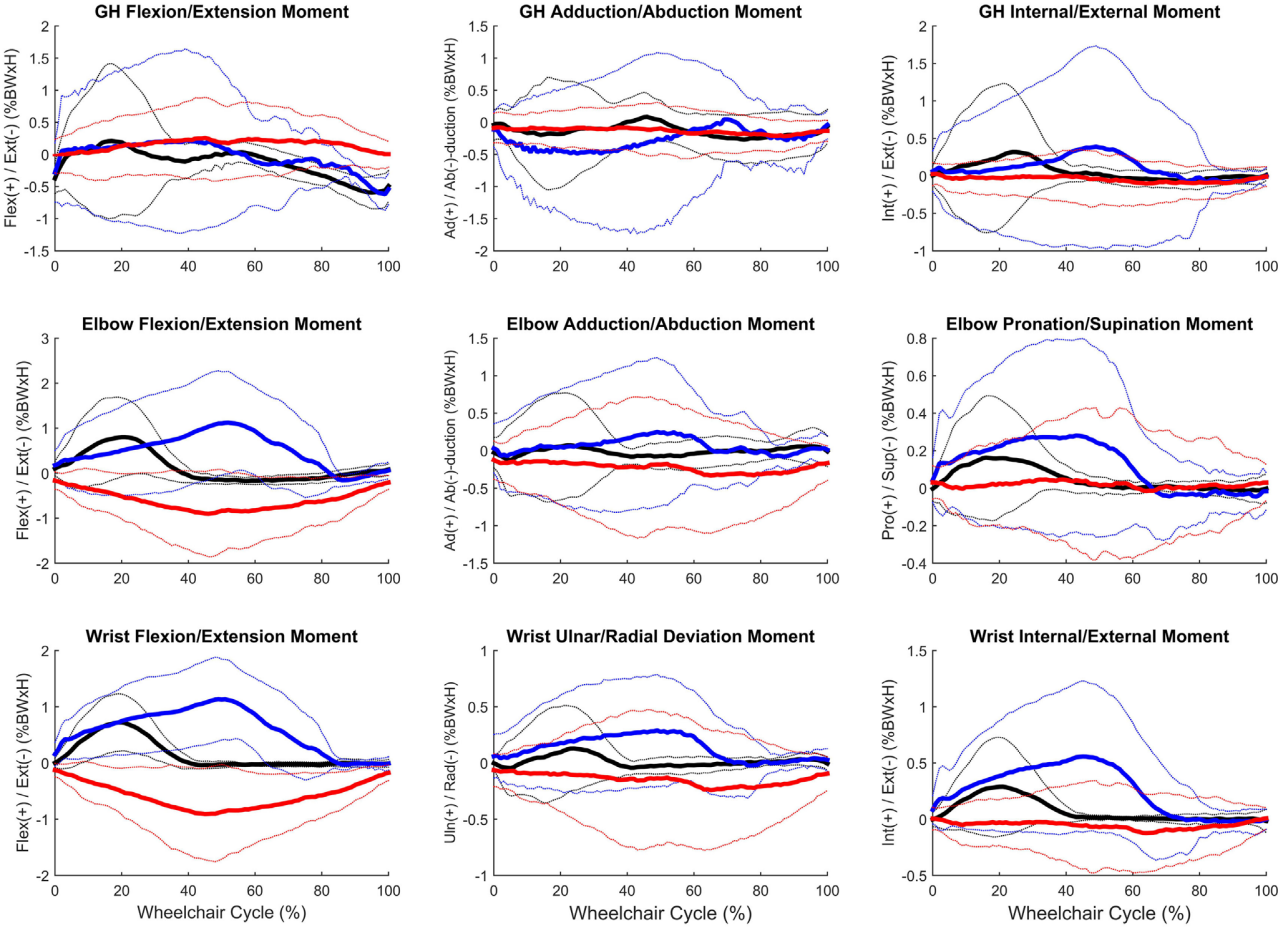
**Group mean (bold) and ±1 SD for the glenohumeral joint moments (top row), elbow joint moments (middle row), and wrist joint moments (bottom row) during the steady-state propulsion (black), start stroke (blue), and stopping stroke (red)**. All moments are normalized to percentage of body weight multiplied by height (% BW × H).

**Figure 10:**
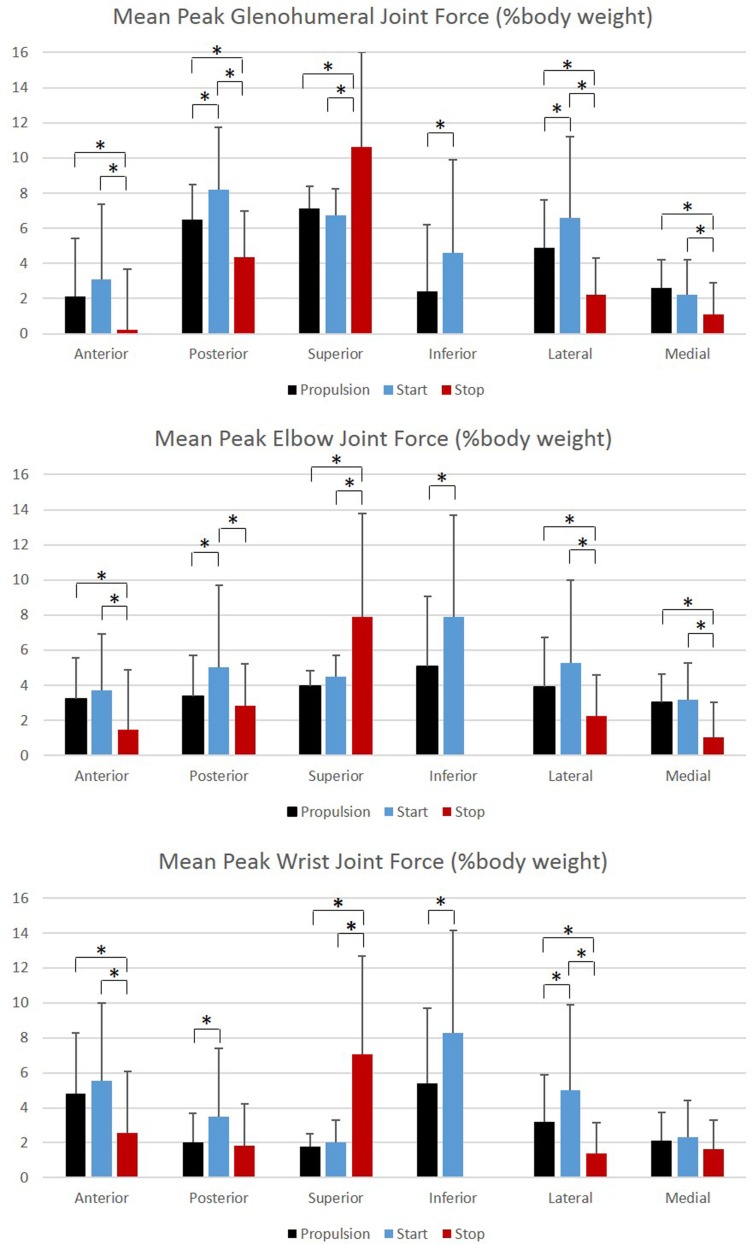
**Group mean peak joint forces (% BW) for the glenohumeral, elbow, and wrist joints during each functional mobility task, propulsion (black), start (blue), and stop (red)**. One SD is represented by the thin vertical bar. Tasks connected by an asterisk are statistically significantly different (*p* < 0.01).

**Figure 11:**
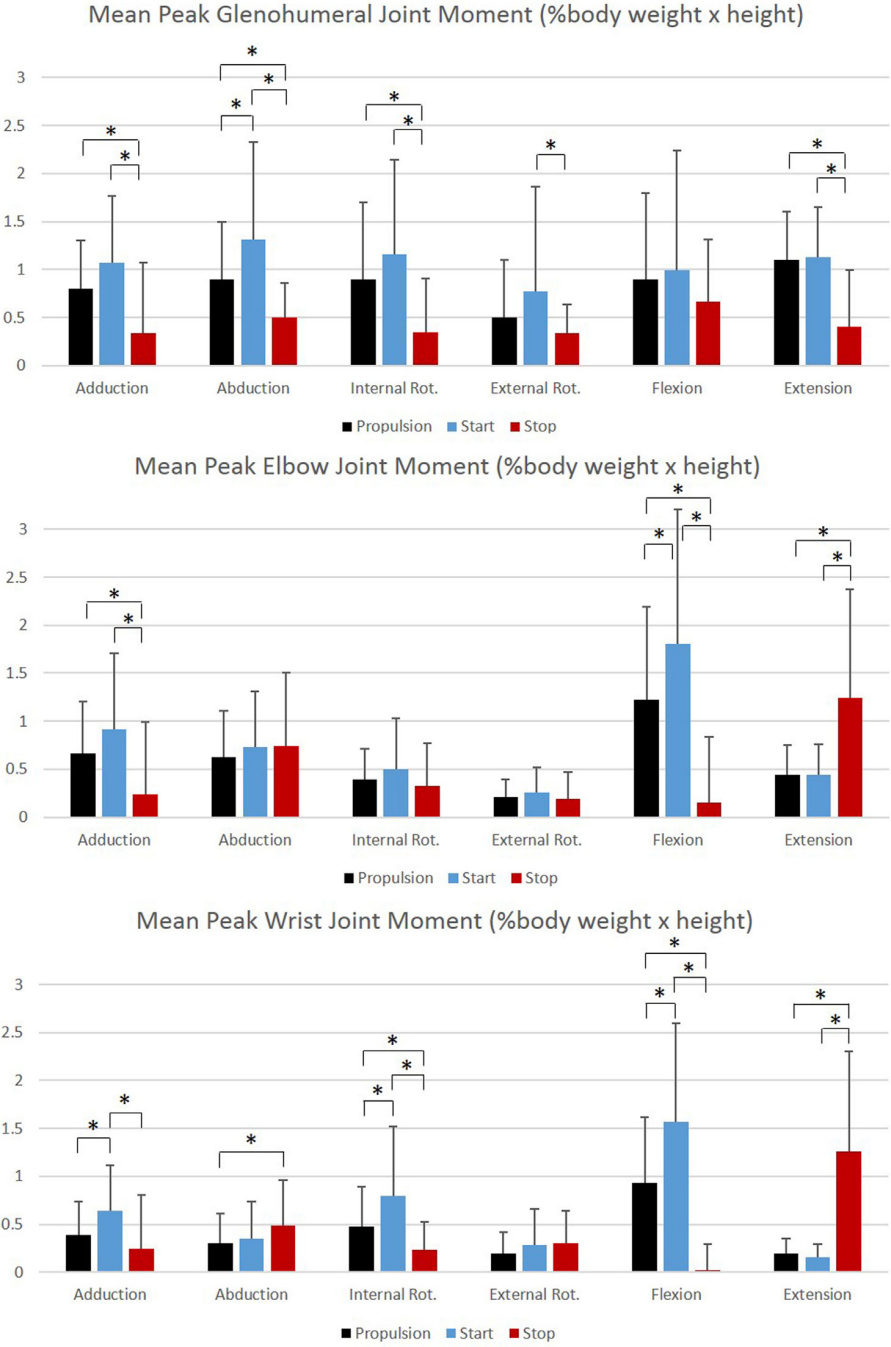
**Group mean peak joint moments (% BW × H) for the glenohumeral, elbow, and wrist joints during each functional mobility task, propulsion (black), start (blue), and stop (red)**. One SD is represented by the thin vertical bar. Tasks connected by an asterisk are statistically significantly different (*p* < 0.01).

Group mean joint forces and moments were characterized three-dimensionally over the wheelchair stroke cycle for the propulsion, start, and stop tasks. The mean and ±1 SD joint forces and moments for the GH, elbow, and wrist joints are displayed in Figures 8 and 9, respectively. The mean peak joint forces (Figure 10) and mean peak joint moments (Figure 11) over the wheelchair stroke cycle were also calculated. Statistically significant differences (*p* < 0.01) in mean peak joint forces and moments among tasks were identified and are depicted in Figures 10 and 11.

### Propulsion stroke patterns

**Figure 12:**
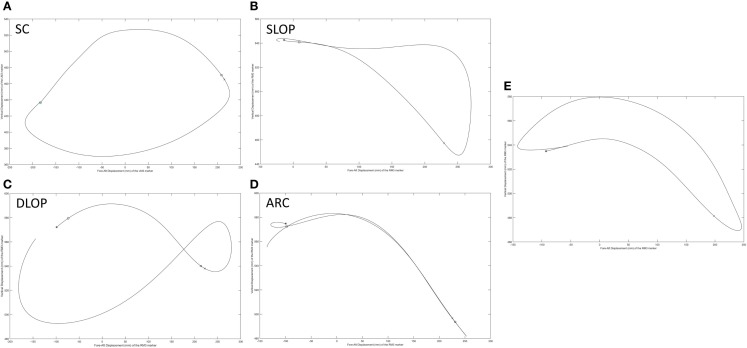
**Representative stroke patterns observed by individuals during the propulsion task**. **(A–D)** Correspond to the four patterns previously identified and classified in adult users: **(A)** semicircular (SC), **(B)** single looping over propulsion (SLOP), **(C)** double looping over propulsion (DLOP), and **(D)** arcing (ARC). **(E)** Does not appear to readily fall into one of these categories.

The stroke patterns utilized during the propulsion task were analyzed qualitatively. While it is currently recommended for adult manual wheelchair users to use the semicircular stroke ­pattern during propulsion (Boninger et al., 2005), each of the four stroke patterns that have been identified and classified in adults (Shimada et al., 1998; Boninger et al., 2002, 2005) were used during propulsion within this pediatric population. In Figures 12A–D, each depict one representative stroke cycle from four different subjects, which clearly identifies with one of the four categories of adult stroke patterns. However, there were also some stroke patterns utilized by the children that do not appear to be properly represented by one of the four current adult classifications, an example is seen in Figure 12E. While this pattern follows the current definition of the single looping over propulsion pattern, “identified by the hands rising above the hand rim during the recovery phase” (Boninger et al., 2002), when comparing it to the typical depiction of adult single looping over propulsion pattern, Figure 12B, the two patterns have strikingly different features, particularly in the later stages of the recovery phase prior to hand contact. Of additional interest is that multiple subjects used more than one stroke pattern throughout the propulsion task trials. Some subjects used different patterns between trials, and some used two or more stroke patterns within the same trial. Therefore, a primary stroke pattern was not evident.

### Pain and health-related quality of life

One individual reported pain, which was minimal (15 on a scale of 0–100). Mean physical component summary scores (PCS) and mental health component summary scores (MCS) acquired with the SF-12 were 44.3 (6.4) and 56.3 (8.2), respectively (normal = 50), indicating lower than normal physical health and higher than normal mental health in this population.

## Discussion

This work provides a unique characterization of joint dynamics and clinical outcomes during pediatric manual wheelchair propulsion, start, and stop tasks. This work is the first of its kind to quantify upper extremity wheeled biomechanics during functional tasks in children. Our group led efforts to investigate pediatric wheelchair propulsion (Schnorenberg et al., 2014; Slavens et al., 2015); however, functional tasks should also be considered. Sonenblum et al. (2012) found that manual wheelchair users were wheeling for only about 10% of the time they spent seated in their wheelchairs per day. Additionally, Cooper et al. (2008) determined that children completed 167 start/stop tasks/1000 m traveled in a day, with an average daily distance of 1600 m, thus, children are completing over 250 start and stop tasks a day, on average. Due to these findings, functional tasks, such as starting and stopping, are presented here. These tasks may be more challenging than propulsion and it is important to understand the joint demands during these functional tasks for improved rehabilitation and treatment planning. Our work is the first to use quantitative methods for determining pediatric joint kinematics and kinetics during functional manual wheelchair mobility, pain, and function. The results of our findings have implications for a comprehensive approach to evaluating pediatric wheelchair mobility.

Overall, the GH joint displayed the largest ROM of 47° (flexion–extension) during the start task and the largest force of 10.6% body weight in the superior direction during the stop task. The elbow displayed the largest peak moment of 1.8% body weight × height in flexion during the start task. Propulsion, starting, and stopping tasks proved to be very different biomechanically, which suggests that clinicians should consider all three tasks when developing rehabilitation protocols and strategies for improving long-term health. GH, elbow, and wrist joint ROMs, were significantly smaller in all three planes, between the propulsion and stopping tasks, and between the starting and stopping tasks. Thus, propelling and starting a wheelchair utilize similar motion demands and magnitudes of the GH, elbow, and wrist joints, while stopping a wheelchair is significantly different. When analyzing the thorax, sternoclavicular, and acromioclavicular joints, there were significant joint ROM differences among all tasks. The start task had the largest ROM amongst the three tasks for all three joints in all planes of motion; however, was only significantly larger in the sagittal plane of the thorax and acromioclavicular joints, and the transverse plane of the sternoclavicular joint. Additionally, the start task ROM was significantly greater than the propulsion ROM in the coronal and transverse planes of the thorax and the coronal plane of the sternoclavicular joint. This shows that while the distal upper extremity joints (GH, elbow, and wrist) are similar between propulsion and start, the body must employ more of the proximal upper extremities (thorax, sternoclavicular, and acromioclavicular) when starting the manual wheelchair. Overall, the start task demands the largest ROM, which is expected due to the nature of beginning movement of the wheel and overcoming inertia. Once the wheel is in motion, as during propulsion, less ROM is needed to keep the wheelchair moving. ROM during starting and stopping is significantly different between tasks in the sagittal plane of all joints, which is also the plane in which the greatest amount of movement occurs during manual wheelchair use.

Peak joint forces and moments provide insight to joint demands and potential risk for injury and overuse. We have successfully quantified upper extremity joint forces and moments during wheelchair propulsion, starting, and stopping. These dynamic tasks were found to be significantly different from one another for GH, elbow, and wrist joint kinetics. All tasks were significantly different from one another for the posterior and lateral GH joint forces and the lateral wrist joint force. Propulsion and starting proved to be significantly different from stopping for all GH joint forces, with only the superiorly directed force greatest during the stop task. The start task demanded the largest amount of force at the GH, elbow, and wrist joints in all planes and directions, with the exception of superior force. While the stop task generally had the lowest joint forces, it had the statistically highest superior joint force across all tasks for all three joints. Additionally, the stop task had the largest overall mean peak force, at 10.6% BW superiorly directed, as well as high superiorly directed joint forces for the elbow (7.9% BW) and the wrist (7.1% BW) joints. We can deduce that subjects placed their hands anteriorly and low on the wheelchair handrim when applying braking grasps, resulting in a pulling of the arm and the resulting high superior joint forces. As quantified here, large amounts of tension are placed on the GH, elbow, and wrist joint during stopping and large amounts of compression force act on the joints during starting. Clinically interesting, propulsion often demonstrates smaller joint force demands than starting or stopping tasks. Despite this, most research has been focused on propulsion. This reiterates the importance of understanding functional wheelchair mobility tasks and their impact on joint force demands. When designing rehabilitation protocols, all functional tasks should be taken into consideration. Propulsion alone should not be the only mobility task considered for wheelchair users when assessing and planning rehabilitation. Particular concern arises with functional tasks since larger joint forces and moments occur during these tasks as compared to propulsion. Further research is warranted to determine the effect of functional tasks on muscle and soft tissue of the shoulder, elbow, and wrist.

Largest joint moments occurred during flexion of the GH, elbow, and wrist joints. When examining the joint moments, they were the highest during the start task. This also supports the notion that the start task may be the most demanding of the tasks. Significant differences among all tasks were seen during GH abduction, elbow, and wrist flexion, and wrist internal rotation moments. Extension moments were significantly different in all joints between propulsion and stopping and starting and stopping. Large variability should be noted, particularly during flexion and at the GH joint.

We successfully identified multiple stroke patterns in this pediatric group of wheelchair users with SCI. In addition to the standard four patterns displayed by adults (i.e., semicircular, ARC, single looping over propulsion, and double looping over propulsion), we also identified a pattern which may require its own classification. Additionally, within subject variability was observed, with some subjects altering their propulsion pattern between and within propulsion trials. Pediatric stroke patterns and the demonstrated variability in movement should be further investigated to determine the most appropriate patterns for particular ages of users, tasks, environments, and levels of injury. Furthermore, additional research is warranted to determine if pediatric subjects should be trained differently than adults. Given these initial findings, it may be beneficial for pediatric subjects to use different stroke patterns than adults as well as a variety of stroke patterns to decrease pain and risk of injury over the lifespan.

The VAS was applied in the study since it serves as the standard outcome tool for clinical pain assessment at Shriners Hospital for Children – Chicago. One subject reported pain, which was minimal. This alludes to the idea that pain has either not yet developed in this group of participants or that the high variability in joint dynamics, as quantified here, is serving as protection to the joints. If so, these movement patterns and variability should be investigated to determine if they could be utilized long term into adulthood to minimize the risk of future pain and injury. Correlation of pain with biomechanical metrics and clinical history (e.g., time since injury and level of injury) is suggested. Further research is underway with a larger population to address these questions. The results of this work also support investigating additional pediatric pain assessment tools that may be more sensitive to upper extremity joint pain or pain during manual wheelchair mobility.

Mean physical health scores (PCS) and mental health scores (MCS) acquired with the SF-12 were 44.3 and 56.3, respectively (normal = 50), indicating lower than normal physical health and higher than normal mental health in this population. Additional outcomes measures are suggested for future assessment of health-related quality of life and correlation with biomechanical and clinical history data.

Although much work has been done for adult wheelchair mobility, there has been limited research on pediatric wheeled mobility, much less on functional tasks. Morrow et al. (2010) investigated intersegmental GH joint demands during functional tasks in adult manual wheelchair users with SCI and noted only GH joint external rotation and extension moments to be greater during starting than propulsion and found no differences among the tasks for GH joint forces. The results, we have found, suggest differences occur between children and adults, which may be attributed to musculoskeletal development and maturation. We believe children should be investigated separately and more comprehensively than adults with additional consideration for musculoskeletal developmental changes, environmental influences, wheelchair size, and strength (Boninger, 2002). We have found that the variability of manual wheelchair propulsion patterns in the pediatric population is quite significant, which may be advantageous in reducing cumulative upper extremity joint demands and pain. Research is in progress, exploring differences in the biomechanics of task performance between children and adults.

### Future directions

This work was the first of its kind to investigate the biomechanics of wheeled mobility tasks in a pediatric population. A larger population is warranted to fully understand the correlation among biomechanics, upper extremity joint pain, function, and health-related quality of life. Work is currently underway to elucidate the relationships amongst these areas with a larger population of pediatric manual wheelchair users. This knowledge will ultimately lead to improved clinical decision-making and rehabilitation paradigms.

Furthermore, evaluation of pediatric wheelchair mobility is essential to determine biomechanical, functional, and joint integrity differences between children and adults. Our work demonstrates that children perform highly variable movement patterns during propulsion, start, and stop tasks, some patterns of which are unlike those classified in adults. A comparison of pediatric and adult biomechanical variability may prove to be essential for improving the health and quality of life of manual wheelchair users. The large variability of joint dynamics (motions, forces, and moments) characterized in this study may relate to age, level of injury, or lack of pain presented by this pediatric population. Additionally, we believe that using a variety of stroke patterns may serve as overuse protection for the shoulder. Additional research directions include determining the rotator cuff muscle activations and forces, which will attempt to clarify the underlying musculoskeletal and tissue effects from pediatric wheeled mobility. Further research is underway to address these questions in a larger population of pediatric manual wheelchair users. The insight gained from this research has the potential to impact pediatric manual wheelchair training, usage, and rehabilitation guidelines.

## Conclusion

Biomechanics of functional manual wheelchair mobility were quantified in children with SCI. Overall, propulsion, starting, and stopping tasks during manual wheelchair use were significantly different biomechanically. Starting a wheelchair appears to be the most demanding task on the upper extremity, while stopping appears to be the least demanding task. However, due to the unique biomechanical demands of each task and patient, clinicians should consider all functional tasks when planning rehabilitation treatment and longer-term mobility strategies. This work also infers that pediatric manual wheelchair users with SCI are different from adult manual wheelchair users and require rehabilitation tailored to their specific needs.

## Conflict of Interest Statement

The authors declare that the research was conducted in the absence of any commercial or financial relationships that could be construed as a potential conflict of interest.

## Abbreviations

3-D, three-dimensional; ARC, arcing; DLOP, double looping over propulsion; FIR, finite impulse response; GH, glenohumeral; LMM, linear mixed models; MR, magnetic resonance; RMS, root mean square; ROM, range of motion; SC, semicircular; SCI, spinal cord injury; SF-12, Short Form 12 Health Questionnaire; SLOP, single looping over propulsion; UE, upper extremity; UEs, upper extremities; VAS, Visual Analog Scale.
